# Correspondence

**Published:** 1868-09

**Authors:** I. A. Salmon

**Affiliations:** Boston


### CORRESPONDENCE.

BOSTON, APRIL 30, 1868.

DR. TAFT.—I wish to correct a mistake made in the February number of the REGISTER, by which I was given the credit of contributing an article on the use of Oxy-Chloride of Zinc. I know not how my name happened to be used, but the credit belongs to Dr. T. B. Hitchcock. I should have made this correction before, but did not notice the article until my attention was called to it a short time since.

I fully endorse the entire article, having used the oxy-chloride for nearly three years with very great success.

I find it the most reliable agent for capping exposed pulps I have ever known, and use it with great confidence even when there is large exposure, and if the pulp is in a healthy condition, even though it may have bled quite freely. Very great care should be used in mixing the preparation that it may be of just the right consistency to *flow* over the exposed portion of the pulp *without the least pressure.* It should be mixed about as thick as cream, but not so thin as to run. If a piece of linen is used it should be coated sufficiently to cause the surface to be somewhat convex, and one edge brought in contact with the dentine near the pulp and carefully allowed to be drawn down to its place by the attractive force of the paste as it flows over the pulp; then a little prepared flax rolled *very loosely*, carefully laid in contact with it

(*without pressure*) to absorb a portion of chloride and facilitate the hardening process which will occupy about ten minutes, when a gold filling can be introduced immediately, with perfect safety in all cases where the system is possessed of the ordinary degree of recuperative power.

I have not kept a record, and cannot state the number of cases I have treated in this manner, but will state the result in one of the most doubtful.

April 18, 1866, Miss D. applied to me to fill the approximal cavities of the left lateral incisor and cuspid. Found both pulps exposed, the cuspid much the largest exposure, and the point of the pulp actually sloughed away. After removing the dead portion and treating the pulp with soothing, healing applications for a day or two, filled the cavities entirely with oxy-chlo. not having examined a sufficient number of cases to warrant me in filling immediately with gold.

These cavities were not filled permanently until June 3, 1867, when they were found to be in a healthy condition, and to all appearance they still live and enjoy good health, never having given the patient any inconvenience up to the present time.

I have been using the os-artificiel, as a matter of convenience, instead of preparing the oxy chloride myself. I suppose all the plastic fillings are substantially the same, being composed of oxide of zinc principally.

I have been thus particular to state the manner of manipulating for the reason that questions have been asked in reference to these points by letter, and there may still be those who would like to ask the same questions, yet would not care to put any one to the trouble of answering by mail.

Yours Very Truly,

I. A. SALMON.

